# ALDH2 promotes uterine corpus endometrial carcinoma proliferation and construction of clinical survival prognostic model

**DOI:** 10.18632/aging.203605

**Published:** 2021-10-20

**Authors:** Yun-Qian Cui, Yuan Xiang, Fei Meng, Chun-Hui Ji, Rui Xiao, Jia-Peng Li, Zhou-Tong Dai, Xing-Hua Liao

**Affiliations:** 1 School of Bioengineering, Qilu University of Technology (Shandong Academy of Sciences), Jinan 250353, Shandong, PR China; 2 Department of Medical Laboratory, Central Hospital of Wuhan, Tongji Medical College, Huazhong University of Science and Technology, Wuhan 430014, Hubei, PR China; 3 Institute of Biology and Medicine, College of Life and Health Sciences, Wuhan University of Science and Technology, Hubei 430081, PR China; 4 Hubei Province Hospital of Traditional Chinese and Western Medicine, Hubei 430010, PR China

**Keywords:** ALDH2, uterine corpus endometrial carcinoma, miR-135-3p, overall survival, risk score model, bioinformatics

## Abstract

UCEC is one of the three common malignant tumors of the female reproductive tract. According to reports, the cure rate of early UCEC can reach 95%. Therefore, the development of prognostic markers will help UCEC patients to find the disease earlier and develop treatment earlier. The ALDH family was first discovered to be the essential gene of the ethanol metabolism pathway in the body. Recent studies have shown that ALDH can participate in the regulation of cancer. In our research, we explored the expression of the ALDH family in 33 cancers. It was found that ALDH2 was abnormally expressed in UCEC. Besides, *in vivo* and *in vitro* experiments were conducted to explore the effect of ALDH2 expression on the proliferation of UCEC cell lines. Meanwhile, the change of its expression is not due to gene mutations, but is regulated by miR-135-3p. At the same time, the impact of ALDH2 changes on the survival of UCEC patients is deeply discussed. Finally, a nomogram for predicting survival was constructed, with a C-index of 0.798 and AUC of 0.764. This study suggests that ALDH2 may play a crucial role in UCEC progression and has the potential as a prognostic biomarker of UCEC.

## INTRODUCTION

Gynecological cancers include ovarian cancer, endometrial cancer (EC), vaginal cancer, cervical cancer and vulvar cancer [1]. EC is a common gynecological malignant tumor. According to statistics, in 2018, there were more than 380,000 new cases worldwide [2]. In developed countries, EC is the most common gynecological cancer. There are more than 60,000 confirmed cases, more than 10,000 deaths, and the death rate is increasing year by year [3]. However, in recent decades, studies have found that early screening and intervention can significantly reduce the incidence and mortality of EC [4–6]. The above data shows that early screening of patients with a high risk of EC is essential, and the discovery of early diagnosis genes will help prevent the occurrence and development of these diseases.

In traditional research, the discovery of new diagnostic markers and prognostic factors through sequencing has the disadvantages of a long time and high cost. The emergence of bioinformatics brings us new research methods for the rapid discovery of new markers and prognostic factors. Recently, more and more studies have shown that bioinformatics can be used as a reliable tool for cancer research [7–9]. In lung cancer, Zhang uses TCGA to conduct a comprehensive analysis of the prognosis, immune function and immune markers based on TNF family markers and developed a risk survival model based on TCGA samples [7]. Similarly, some studies have found that not all TP53 mutations (mainly referring to missense and nonsense mutations) with the help of bioinformatics can effectively predict the therapeutic effect of patients with lung adenocarcinoma (LUAD) on ICIs [8]. However, Li used multiple sets of bioinformatics in the public database to study the expression characteristics, prognostic value, immune infiltration pattern and biological function of Siglec-15, and verified it in patients [9]. In endometrial carcinoma, Wang developed a six-gene prognostic model to predict overall survival [10]. These research results show that exploring new cancer markers and developing new prognostic tools through public databases are a reliable research method.

The human acetaldehyde dehydrogenase family (ALDH family) has 19 members, including ALDH1, ALDH2, ALDH3, etc. ALDH2 has become a hot spot in current research due to the importance of its function. They were first discovered to play a critical role in the oxidation of ethanol in humans [11]. The alcohol dehydrogenase in the liver cytoplasm first metabolizes alcohol to acetaldehyde, and then the acetaldehyde dehydrogenase in the mitochondria gradually metabolizes acetaldehyde into acetic acid. Finally, it is completely decomposed into water and carbon dioxide in the peripheral circulation. However, more and more recent studies have shown that the ALDH family plays an essential role in the occurrence and development of cancer. In gastric cancer, ALDH1 can be used as a tumor stem cell marker [12, 13]. Moreover, the positive expression of ALDH1A1 is associated with low overall survival time and progression-free survival time [14]. In breast cancer, the citral reduces the growth of breast cancer tumors by inhibiting the breast stem cell marker ALDH1A3 [15]. However, in prostate cancer, contrary to tumor tissues, the expression of matrix ALDH1 improves clinical outcome, and is less frequent in PCa metastases [16]. However, there is no report on the relationship between ALDH expression and clinical in UCEC.

In this study, we comprehensively explained the prognostic significance of ALDH2 in UCEC patients. At the same time, a new 6-factor prognostic risk scoring model was developed based on the expression of ALDH2. Meantime, it was found that overexpression of ALDH2 could reduce the proliferation ability of UCEC through *in vitro* experiments. Finally, it was proved that ALDH2 was negatively correlated with miR-135b-3p in UCEC, and it was experimentally verified that miR-135b-3p bind to 3′UTR of ALDH2. These findings could provide theoretical support for the early diagnosis of UCEC patients and the development of new personalized treatments.

## MATERIALS AND METHODS

### Data collection

Expression data and clinical annotation data of 33 types of cancer patients were downloaded from the TCGA data portal (https://gdc.cancer.gov). Perl software (Version 5.3.2) was used in the 33 types of cancer to sort the data to analyze the data for each patient. The expression profile data of normal human EC is obtained from the GTEx database (http://www.gtexportal.org). All database data were accessed in September 2020.

### Analysis of overall survival

R with survival and survminer packages used the Kaplan-Meier method and Cox regression to analyze survival between different groups. *P*-value of less than 0.05 is considered statistically significant.

### Analysis of different genes

R software with limma package was used to analyze the different genes between the data of each group, and log2 foldchange >0.5 and adjust *P* value <0.05 was used as the standard.

### Analysis of gene and pathway enrichment

To determine the biological processes, cell components, molecular functions and biological pathways of differential gene enrichment, R software with clusterProfiler and org.Hs.eg.db packages were used for gene ontology (GO) enrichment and Kyoto Gene and Genome Encyclopedia The whole book (KGGE) approach analysis [17–19]. At the same time, Gene Set Enrichment Analysis (GSEA) was used to analyze related functions among different groups. The screening criterion is *P* < 0.05.

### Construction of protein-protein interaction (PPI) network

The STRING database was used to construct a protein interaction network of differential genes [20]. The screening criterion was a comprehensive score of ≥0.9. Cytoscape software with cytoHubba and MCODE apps was used to visualize and screen highly critical genes and core sub-networks.

### Model construction and verification

To construct the prognostic risk scoring system for UCEC, univariate Cox regression analysis was used to determine the prognostic gene of DEGs. *P* < 0.05 gene was considered as significant. Then, Lasso penalized Cox regression analysis was used to further select OS-related prognostic genes in UCEC. Finally, UCEC patients in the TCGA database were randomly divided into training sets and validation sets with equal numbers, and univariate Cox analysis was used to regress to establish a risk scoring model gradually. The C-index, the AUC value of the model, is calculated by the validation set and the verification of the Nomo diagram to test the accuracy of the risk score model.

### Tumor immunity relevance of the ALDH2

Regarding the close association between the ALDH2 and immune cells of the UCEC microenvironment. Perl software is used to perform statistical analysis on the data downloaded by TCGA. The CIBERSORT deconvolution algorithm (http://cibersort.stanford.edu/) is used to estimate the proportion of different immune cells in cancer tissues, and the differences between immune cells in UCEC and normal adjacent tissues are analyzed. This tool was developed by Newman et al. [21] and has been verified to successfully quantify the abundance of specific cell types.

### Cell culture

Three different cancer cell lines and 293T were purchased from ATCC (ATCC, USA). The highly differentiated EC cell line ISHIKAWA and the moderately differentiated EC cell line SPEC-2 were cultured with MEM medium (GIBICO, USA) containing 10% fetal bovine serum (BI, Australia), The poorly differentiated EC cell line KLE was cultured in DMEM/F12 (GIBICO, USA) mixed medium containing 10% fetal bovine serum. The 293T cell line and endometrial epithelial cell line hEEC were cultured in DMEM (GIBICO, USA) mixed medium containing 10% fetal bovine serum. All cells are cultured in a 37°C incubator containing 5% CO_2_.

### CCK-8 assay

The cells suspension was inoculated in a 96-well plate and continued to culture for 12 h, 24 h, 48 h, 72 h, and then measured cell proliferation ability with the Cell Counting Kit 8 (CCK-8) Kit (Dojindo, Japan). The absorbance values at OD 490 nm were measured using a plate reader (Biorad, USA).

### Colony formation assay

The cells were seeded in a 6-well plate, and when they were cultured normally to form visible clones, the cells were fixed with 4% formaldehyde, stained with purple crystal (Meilunbio, China) and photographed for analysis.

### Xenograft tumor in nude mice

Nude mice were randomly divided into the Control group and the ALDH2 overexpression group. Nude mice in each group were subcutaneously injected with 0.2 ml of UCEC cell suspension (1 × 10^7^ cells/ml) on the ventral side of the right hind limb. On the 21th day, the nude mice were sacrificed by cervical dislocation, the subcutaneous tumors were completely removed, and weighed with an electronic balance. The Wuhan University of Science and Technology Animal Ethics and Use Committee approved the tumor-forming experiment in nude mice.

### Dual-luciferase reporter assay

3′-UTR of ALDH2 and mutant form were separately subcloned into a pmirGLO (Addgene, USA) vector to establish wt-ALDH2-luc and mut-ALDH2-luc respectively. The miRNA mimic, internal control and Luc plasmid were co-transfected into cells. Then, luciferase activities were detected 48 h after transfection by the Dual-Luciferase Reporter Assay System (Promega, USA).

### RNA Pull-Down

RNA pull-down assay was carried out using Magnetic RNA-Protein Pull-Down Kit (Thermo, USA). The RNA-bound beads were added to the cell nuclear lysate. Then, the eluted proteins were detected by western blot analysis.

### RNA Immunoprecipitation (RIP)

RNA-protein-antibody complexes were captured using Protein A/G (Thermo, USA). RNA was eluted by adding TRIzol directly to magnetic beads and isolated as per the manufacturer’s instructions. cDNA was synthesized using HiScript^®^ II 1st Strand cDNA Synthesis Kit (Vazyme, China) and analyzed by qRT-PCR.

### RNA isolation and qRT-PCR

RNeasy Plus Universal Mini Kit (Qiagen, USA) was used to isolate total RNA from cell lines, and HiScript^®^ II 1st Strand cDNA Synthesis Kit (Vazyme, China) was used to reverse transcribe into cRNA. At the same time, the miRNeasy Micro Kit (Qiagen, USA) was used to isolate miRNA from cell lines, and the miRNA 1st Strand cDNA Synthesis Kit (Vazyme, China) was used to reverse the transcription of miRNA. For the extracted RNA and miRNA, qRT-PCR was performed on the Bio-Rad CFX-96 (Biorad, USA) system using the SYBR Green (Yisen, China) method to determine the relative expression level. β-actin and U6 were used as an endogenous control. The primer sequences of ALDH2 are as follows: ALDH2-F: 5-ATGGCAAGCCCTATGTCATCT-3, ALDH2-R: 5-CCGTGGTACTTATCAGCCCA-3. The synthesis of primers, plasmid sequencing, miRNA reverse transcription sequences, primers and probes are all designed by Ribobio (Ribobio, China).

### Western blot

Western blot assay was performed according to the standard protocol. The ALDH2 antibody was purchased from CST (CST, USA), and the antibody was diluted according to the recommended ratio in the instructions.

### Statistical analyses

Comparisons between groups and normality were performed using R 3.6.3 software. Comparisons were completed using a one-way analysis of variance (ANOVA), two-tailed Student’s *t*-test, non-parametric tests. Kaplan–Meier analyses with log-rank tests and the Cox proportional hazard model were used to analyze for survival. All data are presented as the mean ± SD. Statistical significance was set at *P* < 0.05.

### Ethics approval and consent to participate

All mice experimental procedures and methods were evaluated and authorized in strict accordance with the guiding principles of the Animal Protection and Use Committee of Wuhan University of Science and Technology and accordance with the “Hubei Province Experimental Animal Management Regulations.”

### Availability of data and material

The data generated during this study are included in this article and its supplementary, information files are available from the corresponding author on reasonable request.

## RESULTS

### The expression of the ALDH family in all cancers of TCGA

**Figure 1:**
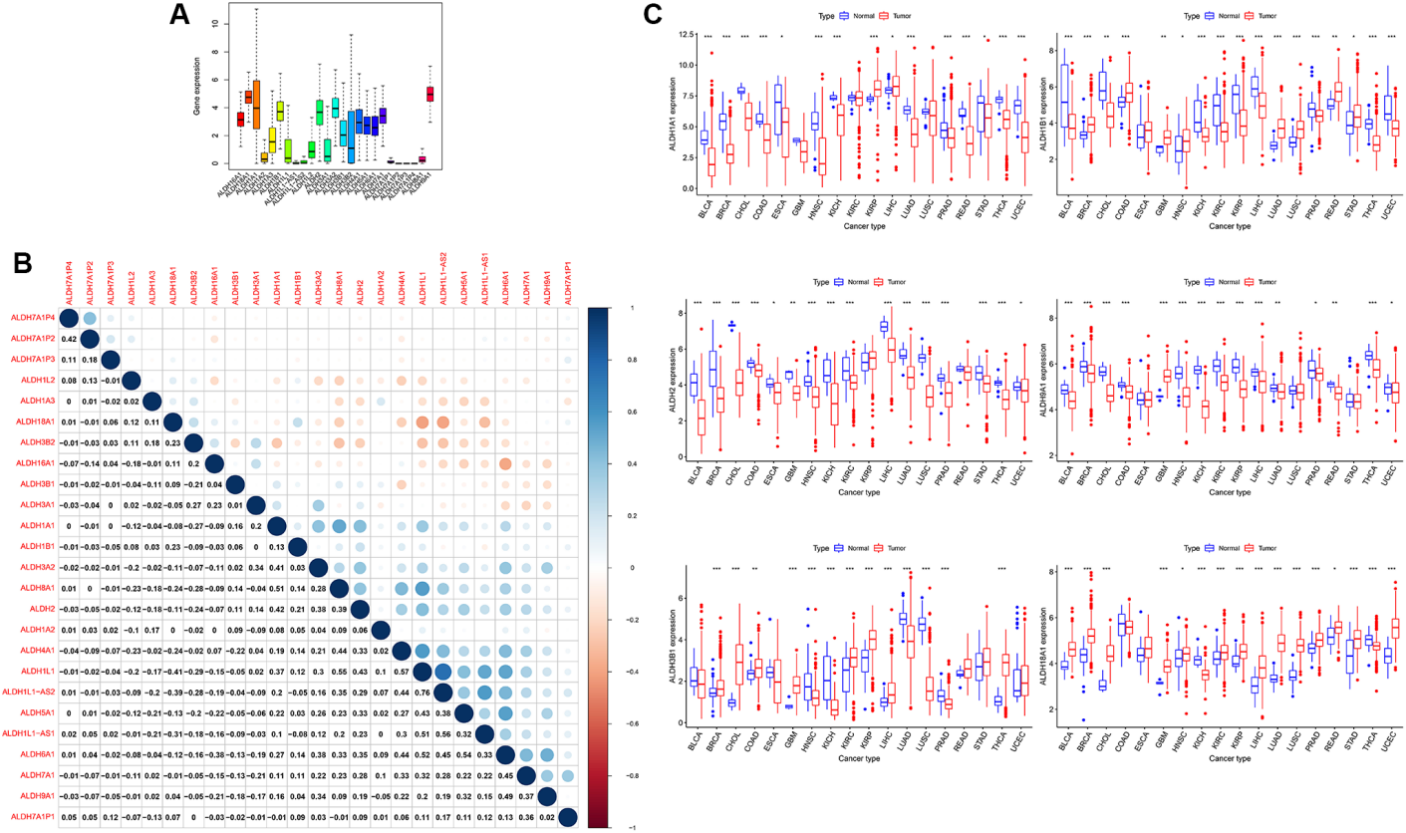
**The expression of ALDH family in all cancers of TCGA.** (**A**) The expression level of patients of ALDH family in 33 types of cancer in TCGA. (**B**) The correlations among the expressions of the ALDH family. (**C**) The relative expression levels of ALDH1A1, ALDH1B1, ALDH2, ALDH9H1, ALDH3B1, and ALDH18A1 in the TCGA database compared with normal tissues. (The number of normal tissue samples less than 3 was hidden). ^*^P < 0.05, ^***^P < 0.001.

Through the TCGA database, the expression data of all ALDH families in all cancers were obtained. As shown in Figure 1, the expression genes of the ALDH family genes in cancer were very different (Figure 1A), and the expressions of the ALDH family genes were highly correlated (Figure 1B). In detail, ALDH9H1, ALDH18A1, ALDH3A2, ALDH1A1, ALDH1B1 and ALDH2 were expressed higher than other ALDH family genes in all cancers. The expression of ALDH2 and ALDH8A1 was highly positively correlated. The first six ALDH family genes were highly expressed in cancer was further analysis. According to the classification of normal tissues and tumor tissues, it was found that the expression of ALDH3B1, ALDH18A1, etc., increased in a variety of cancers, but ALDH9A1 and ALDH2 showed the opposite trend (Figure 1C) in UCEC. Except for ALDH3B1, which was statistically significant, the expression of the other 5 ALDH family members decreased in tumor tissues. Taken together, our data showed that the expression of ALDH was altered in a variety of cancers compared with normal tissues.

### The prognostic significance of the ALDH family

**Figure 2:**
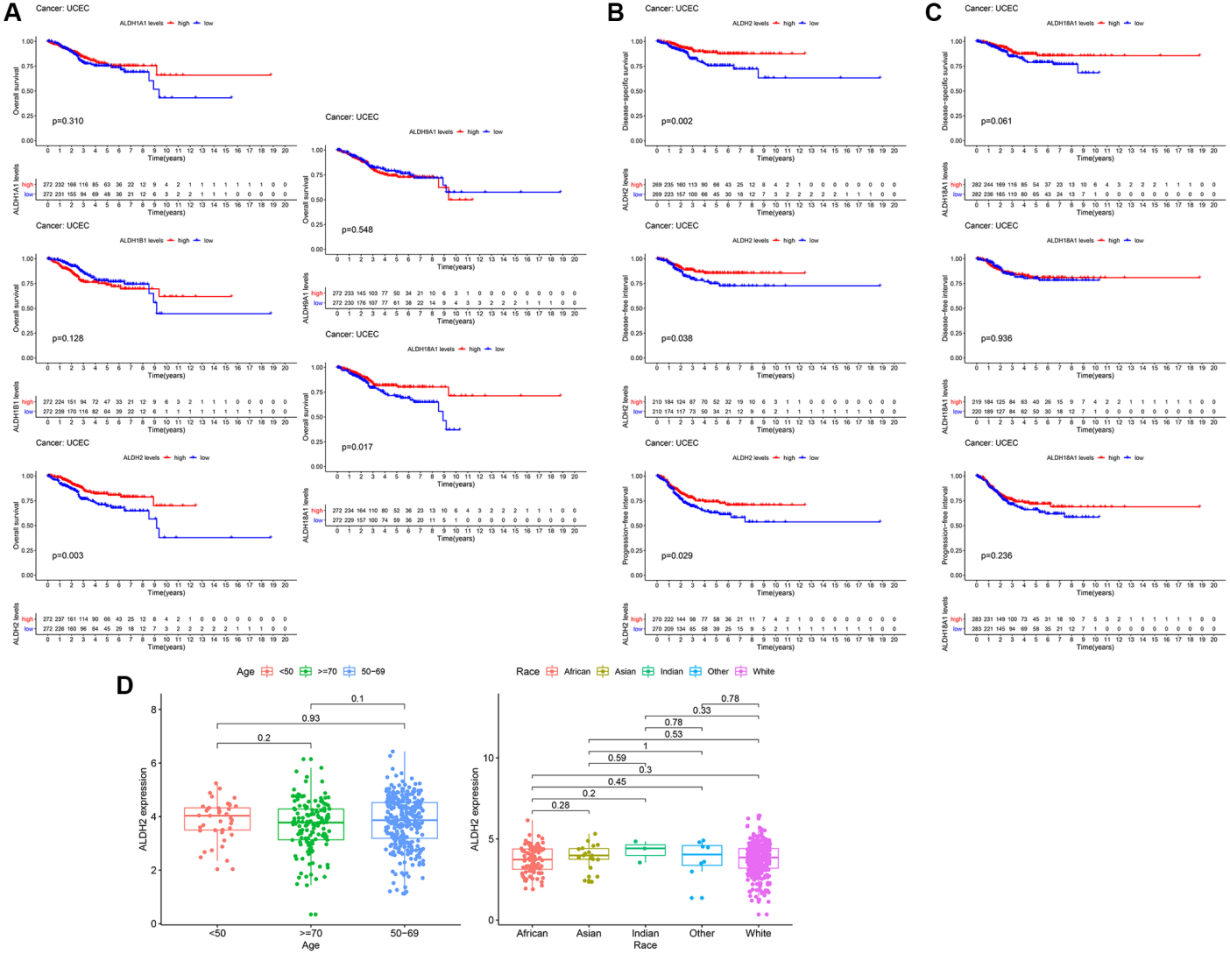
**The prognostic value of the ALDH family.** (**A**) Kaplan–Meier survival curves by OS. (**B**) Kaplan–Meier survival curves by PFI, DSS, and DFI of ALDH2. (**C**) Kaplan–Meier survival curves by PFI, DSS, and DFI of ALDH18A1. (**D**) The relationship between clinicopathological characteristics and the expression of ALDH2.

To further confirm the effect of ALDH family genes that were highly expressed in cancer on UCEC patients, we retrieved the survival information of each patient in the TCGA database, combined with the expression analysis of ALDH family genes. Changes in other ALDH family genes did not affect the overall survival of UCEC patients. Kaplan-Meier analysis results showed that only the expression level of ALDH2 and ALDH18A1 mRNA had a significant impact on the OS of UCEC patients (*P* = 0.003 to ALDH2, *P* = 0.017 to ALDH18A1). Compared with the low expression group, the overall survival time of UCEC patients in the high expression group was significantly prolonged in the lower expression group (Figure 2A). At the same time, the survival of DSS, DFI, and PFI of the low expression group of ALDH2 was the same as the overall survival result, all of which showed a worse survival rate (Figure 2B). For DSS in the ALDH18A1 low expression group, DFI and PFI were not different from the high expression group (Figure 2C). These results indicated that patients in the ALDH2 low expression group were associated with worse prognostic survival in different survival groups. In addition to survival time and survival status, TCGA data also contained complete clinical information of the patient. The results of the clinical analysis revealed that the expression of ALDH2 was not significantly correlated with the age and race of the patients (Figure 2D).

### Analysis of GO, KGGE, GSEA function enrichment

**Figure 3:**
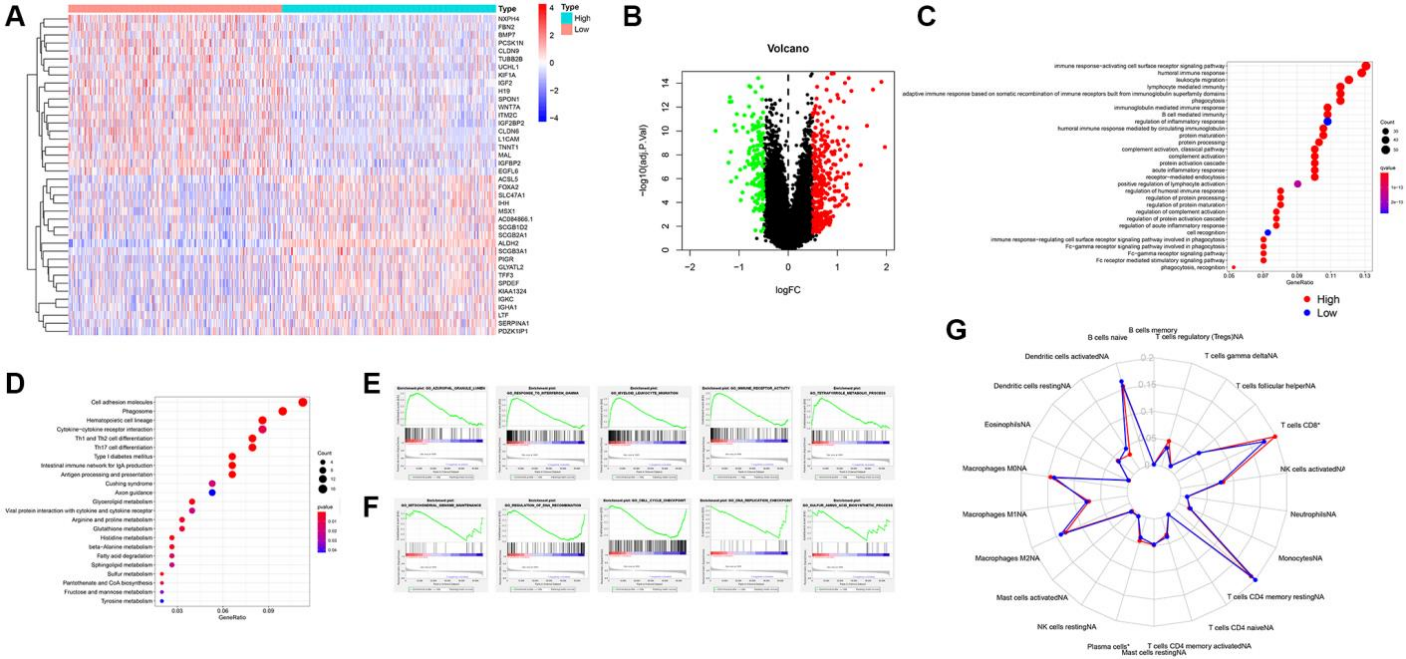
**Analysis of GO, KEGG, GSEA function enrichment.** (**A**) Heatmap showing differential gene expression (FDR < 0.05) between low expression group and high expression group of ALDH2. (**B**) Volcano plot of all differential gene expression analysis. (**C**) GO enrichment analysis. (**D**) KEGG enrichment analysis. (**E**) GSEA enrichment analysis of high expression group of ALDH2. (**F**) GSEA enrichment analysis of low expression group of ALDH2. (**G**) Enrichment scores for 22 immune cell subpopulations based on deconvolution by CIBERSORT between low expression group and high expression group of ALDH2. ^*^P < 0.05, ^***^P < 0.001.

In this study, we downloaded the expression data of all UCEC samples in the TCGA database. The deletion was divided into 2 groups according to the median value and the expression of ALDH2, and the different genes between the groups were analyzed. A total of 477 differential genes were identified. Among them, 322 genes were up-regulated, and 155 genes were down-regulated (Figure 3A, 3B). The enrichment of GO and KEGG helps us understand the potential regulatory functions of these differential genes. The six pathways with the highest correlation found in GO enrichment are, humoral immune response mediated by circulating immunoglobulin, complement activation, classical pathway, complement activation, immunoglobulin mediated immune response, B cell-mediated immunity, and protein activation cascade (Figure 3C). The 6 pathways with the highest correlation of KGGE enrichment are, Cell adhesion molecules, Type I diabetes mellitus, Intestinal immune network for IgA production, Hematopoietic cell lineage, Th1 and Th2 cell differentiation, and Phagosome (Figure 3D). GSEA enrichment showed that the GO processes of the main ALDH2 up-regulated group included Azurophil Granule Lumen, Response to Interferon Gamma, Myeloid Leukocyte Migration, Mmune Receptor Activity, Tetrapyrrole Metabolic Process (Figure 3E). The GO processes of the down-regulation group included Mitochondrial Genome Maintenance, Regulation of DNA Recombination, Cell Cycle Checkpoint, DNA Replication Checkpoint, Sulfur Amino Acid Biosynthetic Process (Figure 3F). The above enrichment results indicated that the alteration of ALDH2 expression might be related to cellular immunity. Therefore, the immune infiltration score of each UCEC patient was calculated by R software with CIBERSORT package. The results showed that patients in the ALDH2 low expression group had lower scores for CD8^+^ T cells and plasma cells (Figure 3G).

### PPI network construction

**Figure 4:**
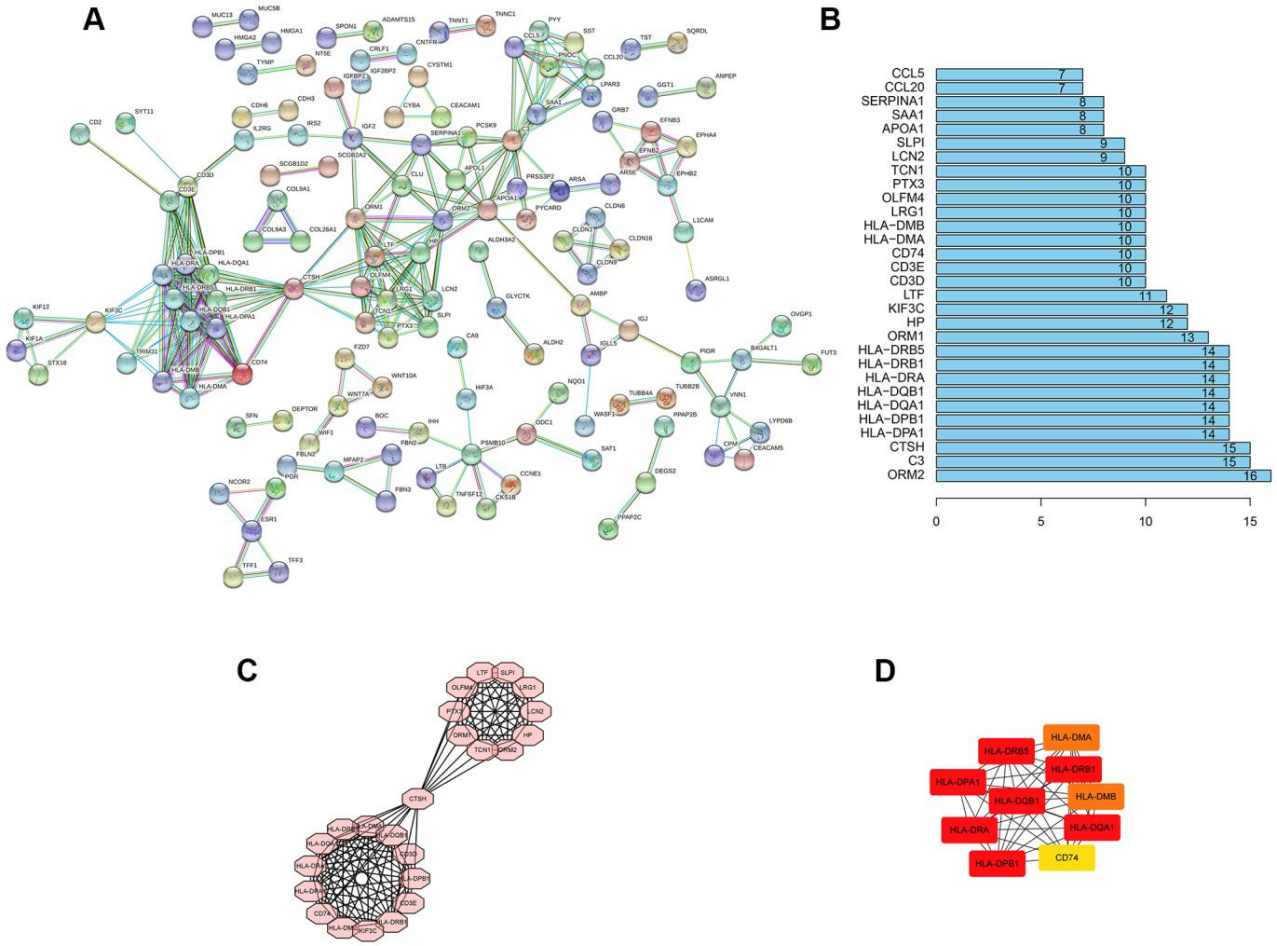
**PPI network construction.** (**A**) PPI network of al DEGs. The individual nodes were hidden. The interaction relationship prediction threshold is > 0.900. (**B**) Top 30 genes with the highest number of nodes. (**C**) The core subnet of the PPI network by using the MCODE app. (**D**) The core gene of the PPI network by using the cytoHubba app.

PPI of DEGs was constructed by using the String network tool. After hiding all the individual nodes, it was found that there were 392 nodes and 295 edges (Figure 4A). Statistics showed that the first six genes with the number of interaction relationships were ORM2, C3, CTSH, HLA-DPA1, HLA-DPB1 and HLA-DQA1 (Figure 4B). At the same time, the MCODE app was used to analyze the most significant interaction modules. The module network consisted of 24 nodes (Figure 4C). In addition, the hub node was calculated through cytoHubba app (Figure 4D). These genes had a potential relationship with ALDH2. It played the role of an oncogene in the occurrence and development of UCEC, which was related to the occurrence, development and prognosis of UCEC.

### The risk score model of UCEC construction

**Figure 5:**
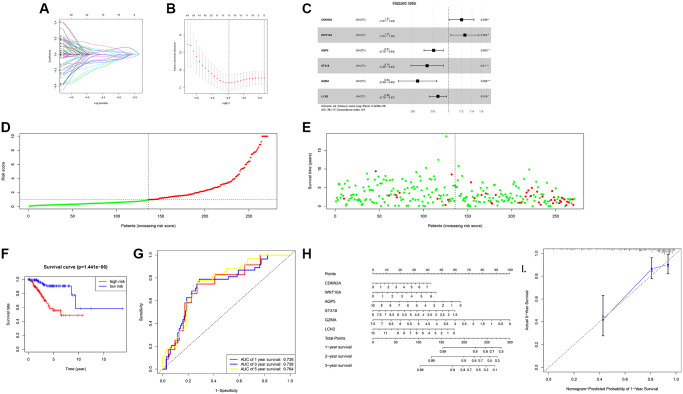
**The risk score model of UCEC construction.** (**A**) DEGs were identified by the LASSO logistic regression model with non-zero coefficients. (**B**) The graph of the relationship between likelihood deviation and log (Lambda). The vertical dashed lines indicate the λ value and the maximum λ value with the smallest error. (**C**) Forest plot of the hazard ratio for OS of parameters. (**D**) Predicted risk of overall survival by the risk score model. (**E**) Scatter plot of UCEC death predicted by risk score model. (**F**) The survival curve of the high expression group and the low expression progenitor in the risk score model. (**G**) ROC curve of risk score model. (**H**) Predicts the OS of patients with the Nomogram. (**I**) Calibration curve of the nomogram in the training dataset.

Our data showed that the high expression ALDH2 group had a better OS, DSS, and PFI (Figure 2). Therefore, a prognostic model based on the overall survival score of ALDH2 expression was constructed. The expression profile of UCEC in the TCGA database was randomly divided into an equal number of training groups and test groups. COX univariate results revealed that a total of 62 genes were significantly related to the overall survival of UCEC patients. Further use lasso regression to screen out 15 genes to prevent the model from overfitting (Figure 5A, 5B). Finally, a 6-factor overall prognostic risk score model was established using COX stepwise regression. The coefficient of risk score model of overall survival was: CDKN2A × 0.18793 + WNT10A × 0.23536 + AQP5 × (−0.21242) + STX18 × (−0.30617) + GZMA × (−0.44271) + LCN2 × (−0.15148). COX multifactorial analysis found that they were all highly significantly related to prognosis (Figure 5C).

Next, use the test group to test the model. The risk scores of 268 UCEC patients in the verification group were shown in the figure (Figure 5D). The state chart showed that the number of deaths in the high-risk group was more (Figure 5E). Moreover, the survival prognosis of the high-risk group was worse (Figure 5F). Besides, the C-index of the model was calculated to be 0.798. The AUC value of 1 and 3 years was 0.738, and the AUC value of 5 years was 0.764 (Figure 5G). These results showed that the model had a better ability to predict. Therefore, a nomogram (Figure 5H) was gained according to the risk score model, and the verification result was shown in Figure 5I.

### Effect of ALDH2 Overexpression on Tumor Progression *in vitro* and *in vivo*

**Figure 6:**
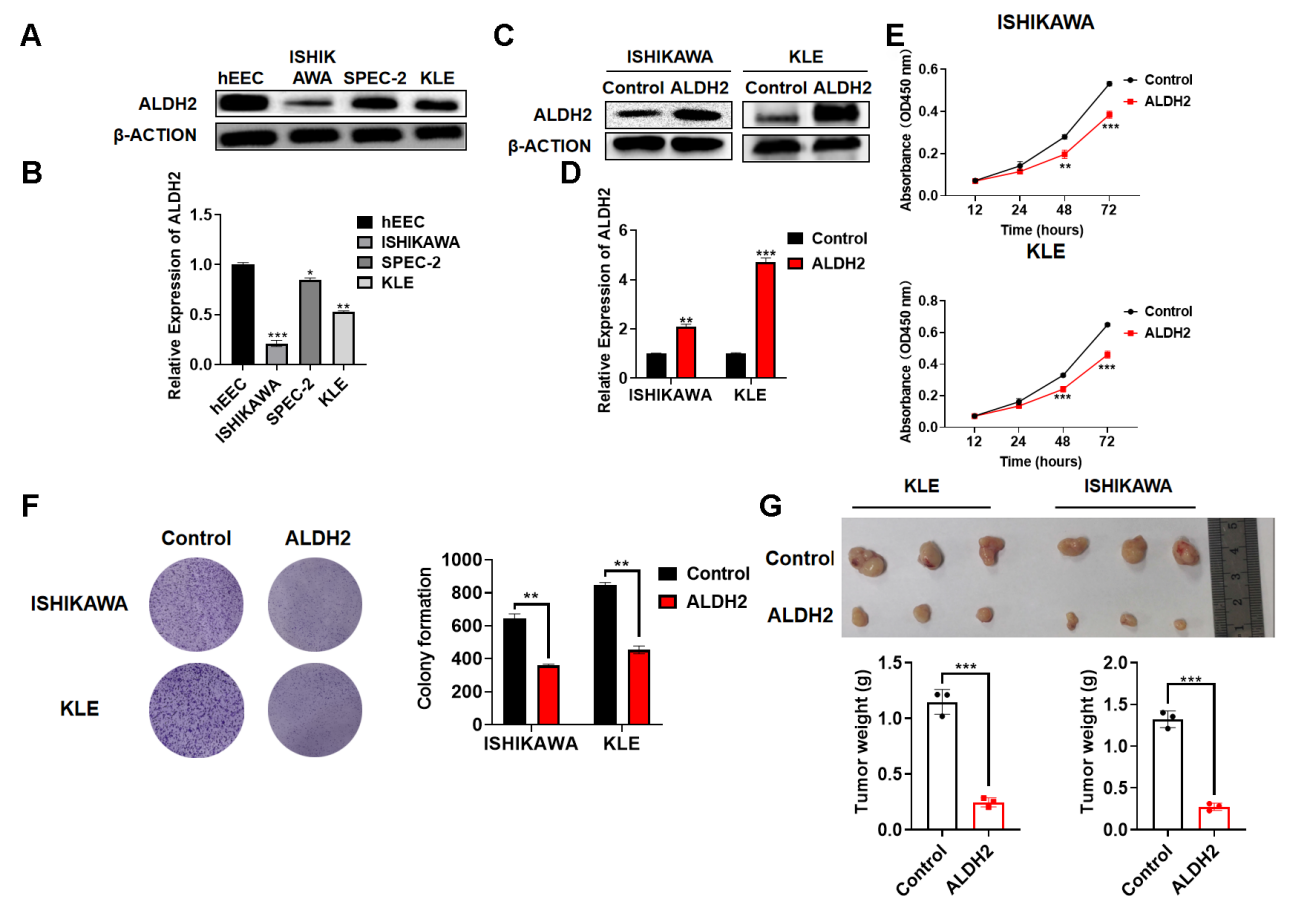
**Effect of ALDH2 overexpression on tumor progression *in vitro* and *in vivo*.** (**A**) Expression of ALDH2 in the UCEC cells by Western Blot. (**B**) Expression of ALDH2 in the UCEC cells by qRT-PCR. (**C**) Overexpression efficiency of ALDH2 by Western Blot. (**D**) Overexpression efficiency of ALDH2 by qRT-PCR. (**E**) CCK-8 assays OD 450 nm. (**F**) Colony formation ability was determined using colony formation assays. (**G**) Tumor xenografts from nude mice subsequent assays. ^*^P < 0.05, ^**^P < 0.01, ^***^P < 0.001.

To determine whether ALDH2 expression was reduced in EC cell lines, it was detected by Western Blot and qRT-PCR. Compared with normal human endometrial epithelial cells hEEC, the expression of ALDH2 was lower in ISHIKAWA, SPEC-2, and KLE (Figure 6A, 6B). In the meantime, a lentiviral plasmid pLKO.1-ALDH2 was constructed to overexpressed ALDH2. The overexpression efficiency was verified by Western Blot and qRT-PCR (Figure 6C, 6D). Next, the relationship between the expression of ALDH2 and the proliferation ability of EC was verified by CCK-8 and colony formation analysis (Figure 6E, 6F). The results showed that restoring the overexpression of ALDH2 reduced the proliferation ability of EC cell lines. Subsequently, a subcutaneous tumor model was used to assess the ability of tumor genesis and growth. As shown in Figure 6G, the tumors in overexpression of the ALDH2 group were significantly smaller than those of the control group (Figure 6G).

### Hsa-miR-135b-3p binding the 3′UTR of ALDH2

**Figure 7:**
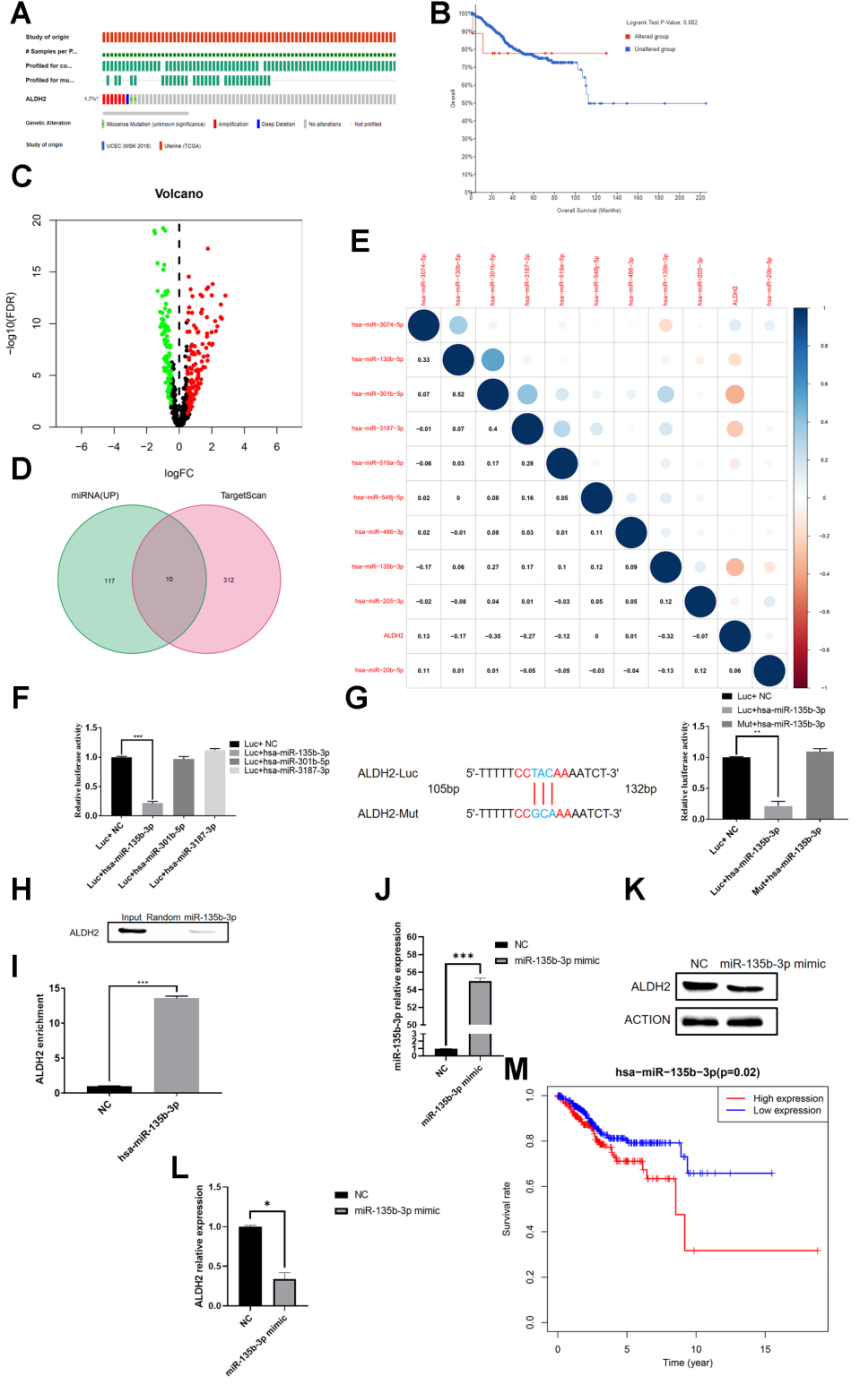
**Hsa-miR-135b-3p binds to the 3′UTR of ALDH2.** (**A**) Gene mutation distribution of UCEC patients detected in TCGA. (**B**) KM survival curve of the mutant group and non-mutation group. (**C**) The volcano plot of miRNAs in UCEC patients with TCGA: Red dots and green dots indicate differentially expressed miRNAs based on the fold change *P* < 0.05. (**D**) Venn diagram of up-regulated miRNA and predicted binding miRNA. (**E**) Correlation analysis between the expression of predicted bound miRNA and the expression of ALDH2. (**F**) Relative Luciferase activity was measured with a dual-luciferase reporter assay. (**G**) Dual-luciferase assay of the mutation group. (**H**) RNA pull-down analysis with ALDH2 antibody. (**I**) RIP assay was further verified for a direct association between hsa-miR-135-3p and ALDH2. (**J**) Overexpression efficiency of hsa-miR-135-3p mimic. (**K**) WB analysis the expression of ALDH2 with overexpression of hsa-miR-135-3p. (**L**) Relative expression of ALDH2 with overexpression of hsa-miR-135-3p by using Image J software. (**M**) Kaplan-Meier survival curve for patients with UCEC (According to the median value of aldh2 expression, it is divided into two groups of high and low expression). ^*^P < 0.05, ^**^P < 0.01, ^***^P < 0.001.

In the previous analysis, the OS of UCEC patients in the ALDH2 low expression group was worse (Figure 2A). Therefore, whether ALDH2 genetic mutations were the cause of the decreased OS in UCEC patients was investigated. Analysis of gene mutation frequency was found that in most patients, it was not the mutation that caused the disappearance of ALDH2 effect (Figure 7A, 7B).

Numerous studies had proved that miRNA could silence gene expression by targeting gene 3′UTR, consequently, it was speculated that ALDH2 might also be regulated by this mechanism. By screening the difference in miRNA between UCEC patients and the normal group, the volcano plot showed that a total of 122 up-regulated miRNAs were screened (Figure 7C). At the same time, the bioinformatics prediction website TargetScan was also visited. As a result, 312 miRNAs were predicted to bind to ALDH2’s 3′UTR. A total of 10 miRNAs were selected from the intersection, and the correlation between their expression and ALDH2 expression was shown in the figure (Figure 7D, 7E). The top three miRNAs with the highest negative correlation were hsa-miR-301b-5p, hsa-miR-3187-3p and hsa-miR-135b-3p.

To verify which miRNA regulated the expression of ALDH2, the Luciferase plasmid of ALDH2 3′UTR was constructed. The dual fluorescein report experiment results showed that only the hsa-miR-135b-3p transfection group had a decrease in relative fluorescence intensity. However, there was no significant difference between hsa-miR-301b-5p and hsa-miR-3187-3p after transfection (Figure 7F). The mutation group showed that the mutation could eliminate the inhibitory effect of hsa-miR-135b-3p on ALDH2 (Figure 7G). Simultaneously, RIP and RNA pull-down found that hsa-miR-135b-3p could enrich the expression of ALDH2, but the control group did not have this phenomenon (Figure 7H, 7I). QRT-PCR showed that after transfection of hsa-miR-135-3p mimic, the expression of hsa-miR-135b-3p was enhanced significantly (Figure 7J), while the results of WB and qRT-PCR showed that the expression of ALDH2 was decreased (Figure 7K, 7L). Besides, TCGA survival data showed that the high expression group of hsa-miR-135b-3p had a lower prognosis (Figure 7M). These results indicated that hsa-miR-135b-3p could down-regulate the expression of ALDH2.

## DISCUSSION

Endometrial cancer is one of the three most common malignant tumors of the female reproductive tract, and it is the sixth most common cancer in women worldwide [2]. With the increase in the average life expectancy of the population and the change in living habits, the incidence of EC has continued to rise and become younger in the past two decades [22]. Although the cause of endometrial cancer is not very clear so far, it may be due to genetics, obesity, or the use of drugs [23]. However, clear results are showing that the cure rate of early UCEC can reach 95% [24]. At present, the early diagnosis of UCEC is usually based on the clinical manifestations of patients, such as postmenopausal bleeding or abnormal serum levels of certain tumor markers, and about 15% of UCEC occur in women without vaginal bleeding [25]. Studies have reported the role of different serological markers in the diagnosis of UCEC, such as carcinoembryonic antigen, carbohydrate antigen-125 and carbohydrate antigen 19–9, but their expression is only up-regulated in 20% to 30% of UCEC patients [25, 26]. Due to the delayed diagnosis, UCEC patients often lose the best treatment opportunity, leading to a higher risk of tumor metastasis and postoperative recurrence, and a poor prognosis [27, 28]. A number of studies have shown that some of the genes that are dysregulated in UCEC patients can be used as biomarkers for their diagnosis [29–31]. Therefore, the development of prognostic markers will help UCEC patients to find the disease earlier and develop treatment earlier.

However, due to traditional experimental methods, the time period is long, and the research and development costs are high. The emergence of bioinformatics has brought us the possibility of studying new prognostic markers. Several research results have shown that bioinformatics is a reliable research tool. In gastric cancer, GDF-15 can be used as a biomarker for gastric cancer [32]; In liver cancer, YTHDF1 expression is elevated in liver cancer patients [33]; In UCEC, Li found that Mammaglobin B is a prognostic marker of UCEC [34]. Similarly, we downloaded the expression data of 33 cancers in TCGA. Extracting ALDH family genes found that the expression of the ALDH family has changed significantly in most cancers. The ALDH family was first discovered to be mainly involved in the process of alcohol metabolism [35]. However, recent studies have found that ALDH family genes can participate in the regulation of cancer. In detail, ALDH1 has been identified as a tumor stem cell marker involved in the development of cancer [36]. As for ALDH2, Li found that restoring the expression of ALDH2 in lung adenocarcinoma can inhibit the migration of lung cancer cells, which was consistent with our findings [37]. We found that ALDH2 expression in most cancers was declined not only in LUAD but also in UCEC. The characteristic information was found that the change of ALDH2 was not significantly correlated with age group and race. Combined with the clinical information of UCEC patients in the TCGA database, it was found that in UCEC, the OS, DSS, DFI and PFI of the low expression group of ALDH2 had worse survival conditions. Bioinformatics enrichment function was found that the changes of ALDH2 participate in cancer mainly through the regulation of immune function. The previous discussion showed that ALDH1 had been identified as a tumor cell marker [36], for instance, Christophe found that ALDH1 cell subsets had higher cancer stem cell characteristics [38], Mohamed identified that the expression of ALDH1 was highly correlated with the expression of Colorectal Carcinoma’s tumor stem cell markers Notch1 and CD44 [39], and we calculated the characteristic value of immune infiltration of UCEC patients through CIBERSORT and found that the change of ALDH2 was highly correlated with CD8+T cells. However, there are no reports about the changes of ALDH2 and the research of cancer stem cells and immune-related research. Our findings provide new ideas for further research on the regulation of ALDH2 on cancer. In addition, we initially explored the relationship between the expression of ALDH2 and the progress of UCEC *in vivo* and *in vitro*. It was found that the expression of ALDH2 was reduced in the three UCEC cell lines ISHIKAWA, SPEC-2, and KLE. At the same time, restoring the expression level of ALDH2 in the cell line could reduce the proliferation ability of tumor cells, and *in vivo* experiments have also verified our findings.

Gene mutation is one of the main factors of gene inactivation. Through the cBioPortal database, it was found that the changes in ALDH2 were not due to genetic mutations. In recent years, a new way of regulating genes non-coding RNA regulating genes has been widely verified. Non-coding RNA is a type of RNA that cannot encode the protein. It has been considered a “junk” transcription product for a long time. However, research in recent years has changed people’s understanding of ncRNA, and more and more studies have focused on ncRNA. It is a class of functional regulatory molecules that can regulate a series of cellular processes, including chromosome remodeling, transcription, post-transcriptional modification, and signal transduction, and plays a crucial role in developmental and disease processes [40]. ncRNA mainly includes micro miRNA, lncRNA, and circRNA, which coordinates their parts and plays an essential role in the complex function regulation network of cells. Mirna is a kind of endogenous non-coding RNA, which is generally composed of 22 nucleotides, It regulates gene expression by binding to the 3′UTR of target mRNA [41]. In 2002, Calin et al. confirmed the presence of miRNA in chronic leukemia cells. The expression of miR-15 and miR-16 is reduced or even missing. This is the earliest direct evidence that the abnormal expression of miRNA is related to tumorigenesis [42]. Subsequently, more and more tumor miRNA chips confirmed that miRNA can indeed regulate the expression of proto-oncogene or tumor suppressor gene. They play an important role in the occurrence and metastasis of a variety of malignant tumors [43–45]. By taking the intersection of the UCEC data, it was found that the expression of miR-135-3p in UCEC was increased, which was negatively correlated with the expression of ALDH2. Therefore, through dual-luciferase reporter gene experiments, RIP, RNA pull-down, it was found that miR-135-3p could target ALDH2. Meanwhile, the expression of miR-135 was up-regulated in NSCLC cells. Silencing miR-135 can inhibit cell viability, migration, and invasion [46]. In lung cancer, overexpression of ALDH2 inhibits the malignant characteristics of lung adenocarcinoma cells, such as proliferation, stemness, and migration, while knocking down ALDH2 increases these characteristics [37]. These results verify this regulatory relationship from the side view.

We found earlier that the low expression group of ALDH2 had a worse survival status. Therefore, we developed a risk score model based on ALDH2 expression to predict the survival of UCEC patients. To construct a risk scoring model, we screened 478 differentially expressed mRNAs from 538 originals in the UCEC dataset in the TCGA database according to the expression of ALDH2. Based on COX regression and Lasso Cox regression model analysis, screening and constructing a prognostic risk scoring model was accomplished. The risk scoring model can divide UCEC patients into high-risk and low-risk groups to predict their prognosis. In addition, whether it is through the time-dependent ROC curve or the C-index of the calculation model, the risk scoring model we build has high predictive sensitivity and performance.

The TCGA database provides complete data related to cancer, including gene editing, mutation information, clinical survival information, and so on. In this study, by using the information provided by the TCGA database, we found that most of the ALDH family genes were abnormally expressed in 33 cancers. Among them, in UCEC, the expression of ALDH2 decreased, and the low expression group had a worse prognosis. except this, we preliminarily discussed how the changes in ALDH2 are involved in the regulation of cancer and preliminarily verified that miR-301a-5p targets the 3′UTR of ALDH2. Finally, a risk score model was constructed, and which has high performance in terms of specificity and accuracy. It provides new ideas and directions for further exploring the mechanism and treatment strategies of UCEC. However, current research still has some limitations.

## CONCLUSIONS

This study analyzed the expression of ALDH2 and its family genes in 33 cancers. And in UCEC, the expression of ALDH2 and the role of survival were explored in detail. It was found that the expression of ALDH2 was reduced in UCEC patients, and the survival status of the low expression group was worse. Meantime, bioinformatics and experimental methods were used to verify the function of ALDH2 in UCEC patients. The results showed that restoring the expression of ALDH2 could inhibit tumor progression, and the expression of ALDH2 was regulated by hsa-miR-135-3p. In addition, a survival model based on the expression of ALDH2 was proposed, whose C-index is 0.798 and AUC is 0.764.
